# Supplementary figures and images for: Monitoring integrated stress response in live Drosophila
Source: bioRxiv. 2023 Jul 14:2023.07.13.548942. Preprint. [Version 1] doi: 10.1101/2023.07.13.548942 (PMC10369977; doi:10.1101/2023.07.13.548942)

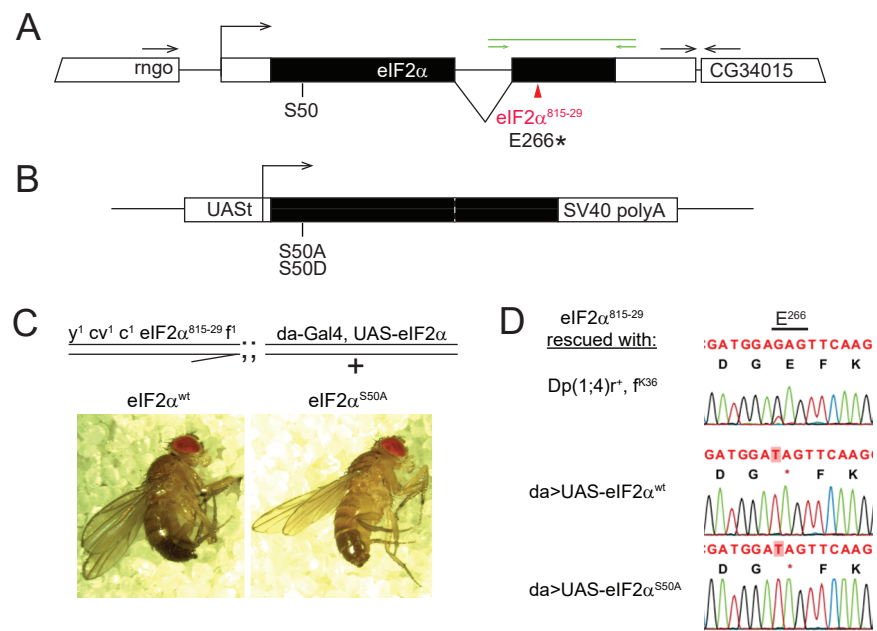

FIG S1

Supplement: Supplement 1 — Fig S1. Validation of UAS-eIF2α constructs. A. Gene structure of eIF2α. The green arrows correspond to the oligos, and the line – to the PCR product that was used to genotype eIF2α815–29 (E266*) mutation. Both oligos anneal in intronic sequences. Thus, the intronless UAS-eIF2α constructs (B) are not recognized by this oligo pair. C. Adult males rescued with UAS-eIF2αwt and UAS-eIF2αS50A driven with da-Gal4 driver. [file media-1.pdf]
